# Massive Localized Lymphedema in an Unreported Location (Retroperitoneum)

**DOI:** 10.1186/s13000-018-0769-x

**Published:** 2018-11-20

**Authors:** Dilek Ertoy Baydar

**Affiliations:** Department of Pathology, Koc University School of Medicine, Topkapi / Zeytinburnu, 34010 Istanbul, Turkey

**Keywords:** Massive localized lymphedema, Lymphedema, Retroperitoneum, Surgery

## Abstract

**Background:**

Massive localized lymphedema (MLL) is a non-neoplastic benign soft tissue lesion that may be confused with sarcomas or other neoplastic proliferations both clinically and morphologically. Most occur in morbidly obese adults on the lower extremities. The objective of this article is to document a case of MLL in the retroperitoneal cavity which is a previously unreported site for this lesion, and to highlight its unusual clinical features.

**Case presentation:**

The patient was a non-obese male who had undergone major abdominal surgery due to bladder extrophy 17 years ago. Abdominal ultrasonography detected a large incidental mass in the right renal sinus during his investigation for nephrolithiasis. The lesion extending from renal pelvis down to pelvis was resected and its histopathological findings were compatible with massive localized lymphedema.

**Conclusions:**

Retroperitoneum has to be added to the list of locations that MLL can be found. Liposarcoma will be a challenging differential diagnosis when the lesion is encountered in an unusual site.

## Background

Massive localized lymphedema (MLL) is a rare pseudosarcomatous lesion afflicting predominantly morbidly obese or obese individuals, first described by Farshid and Weiss in 1998 [1]. It presents as a large painless mass slowly enlarging over many years. The multifocality has been emphasized in a subgroup of cases [2]. Although MLL is a seldom entity in the literature, it is assumed that these tumors will be increasingly encountered in the future due to the rising prevalence of obesity. They have significant potential for confusion with malignancies both clinically and histologically, such as well-differentiated liposarcoma especially when they are deeply localized. This article reports a unique case of MLL that developed in the retroperitoneal cavity of a mildly overweight person, possibly related to his past surgery 17 years ago. This is the first case of retroperitoneal MLL presented in the literature.

## Case presentation

53-year-old man underwent abdominal ultrasonography (USG) during investigation for bilateral renal nephrolithiasis. The patient had normal blood biochemistry, and had no cardiovascular or hormonal disorder. He had been operated 17 years earlier to repair his extrophic bladder by creating an Indiana pouch. His weight was 85 kg.s with a body mass index of 28.7 kg/m^2^. USG showed a hyperechogenic lesion at the fat intensity filling out right renal sinus completely. Computerized tomography scan confirmed the presence of a fatty mass that extended from the renal sinus down to the pelvis cuffing the right ureter throughout its entire length with severe hydronephrosis. Left kidney was unremarkable except a small scar at the upper pole. The presence of high density regions inside the lesion imparted a suspicion for liposarcoma. The resection of the mass with right radical nephroureterectomy was performed.

**Fig. 1:**
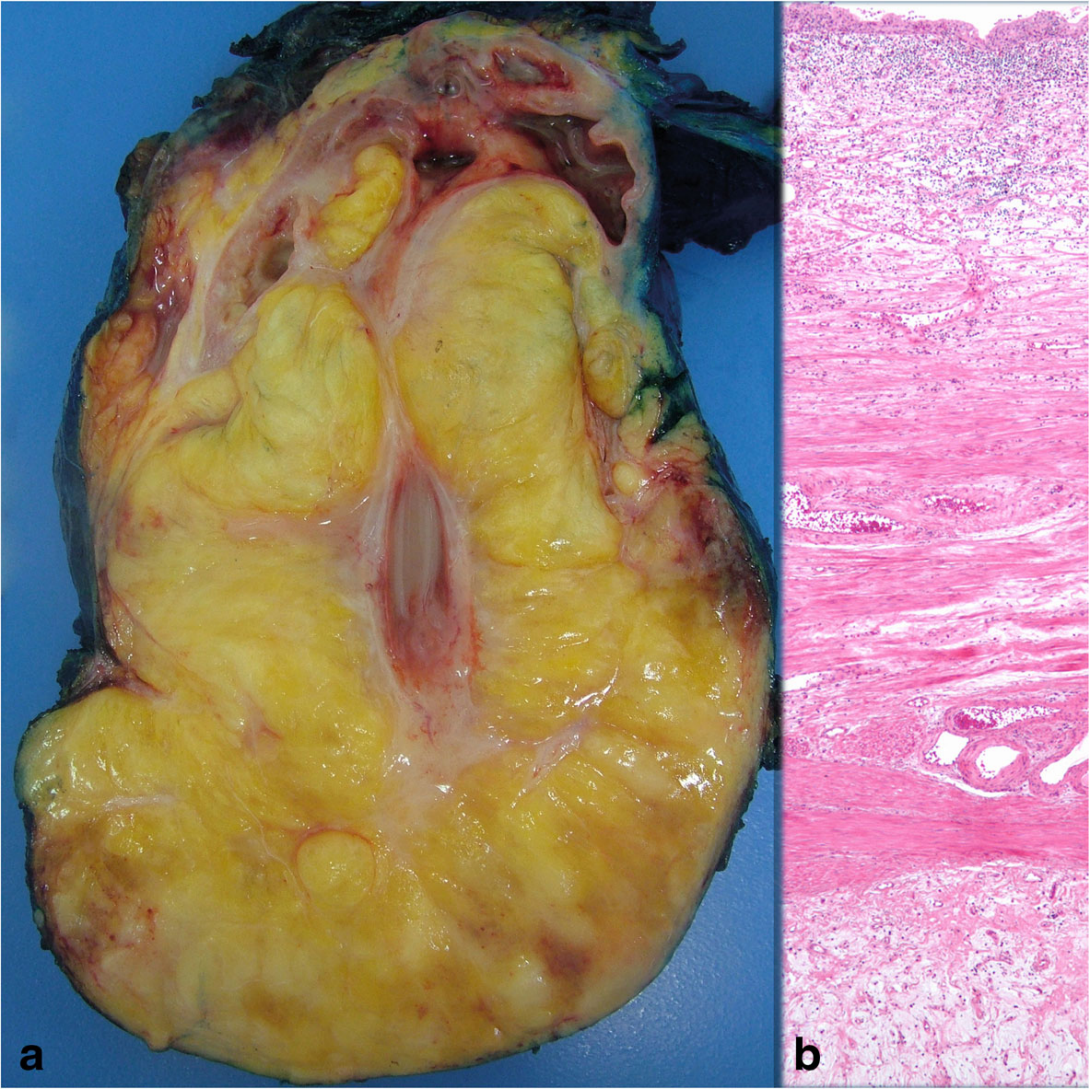
**a** Huge fatty mass occupying renal sinus and entrapping ureter. Kidney was small but hydronephrotic. **b** Thick ureter wall and the surrounding lesion beneath it (H-E × 40)

**Fig. 2:**
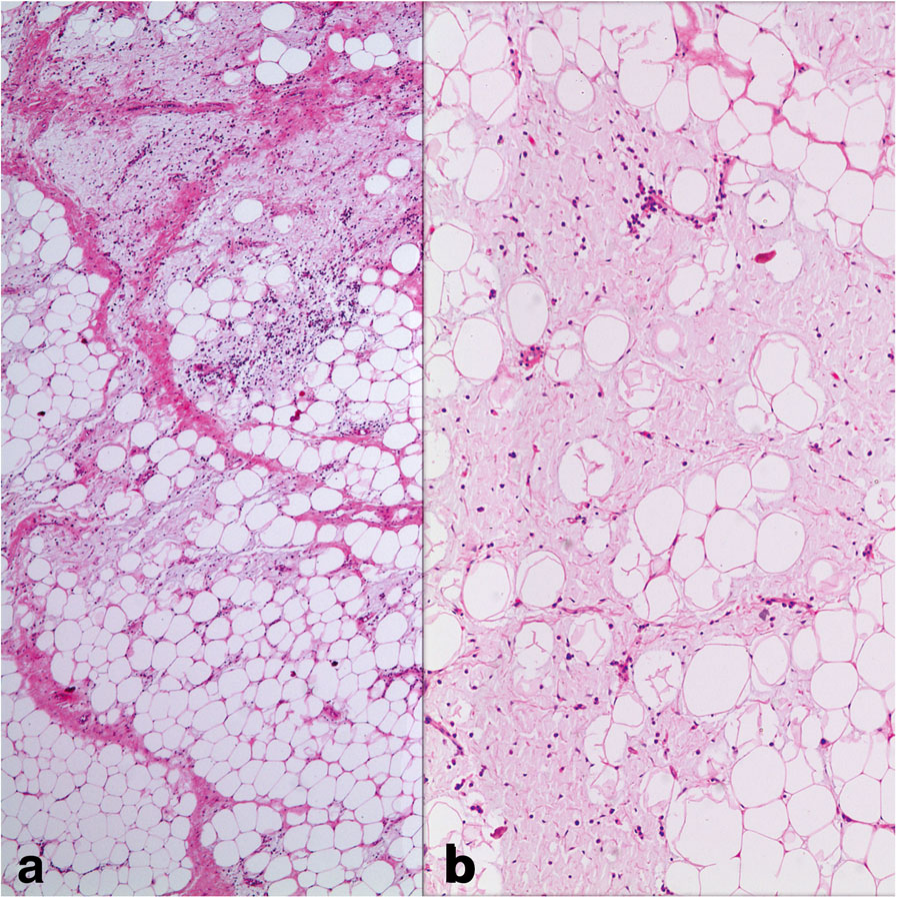
Significant interstitial edema (**a** H-E × 40; **b** H-E × 100)

**Fig. 3:**
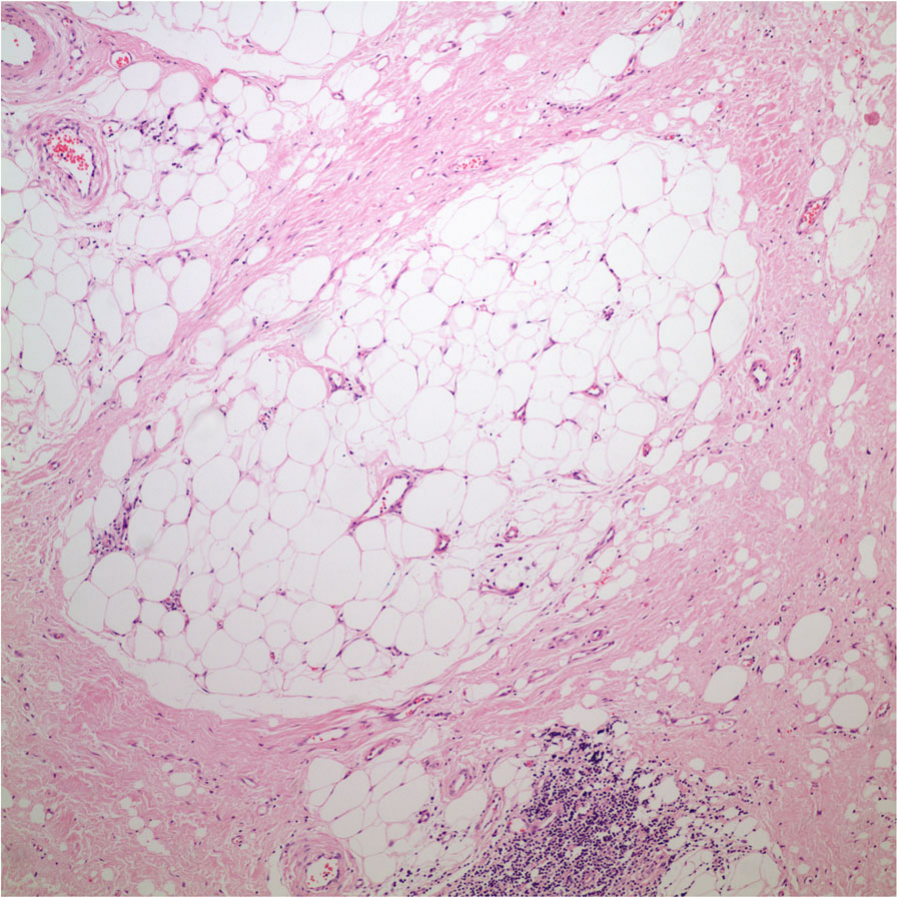
Irregular fibrous bands separating groups of fat cells (H-E × 100)

**Fig. 4:**
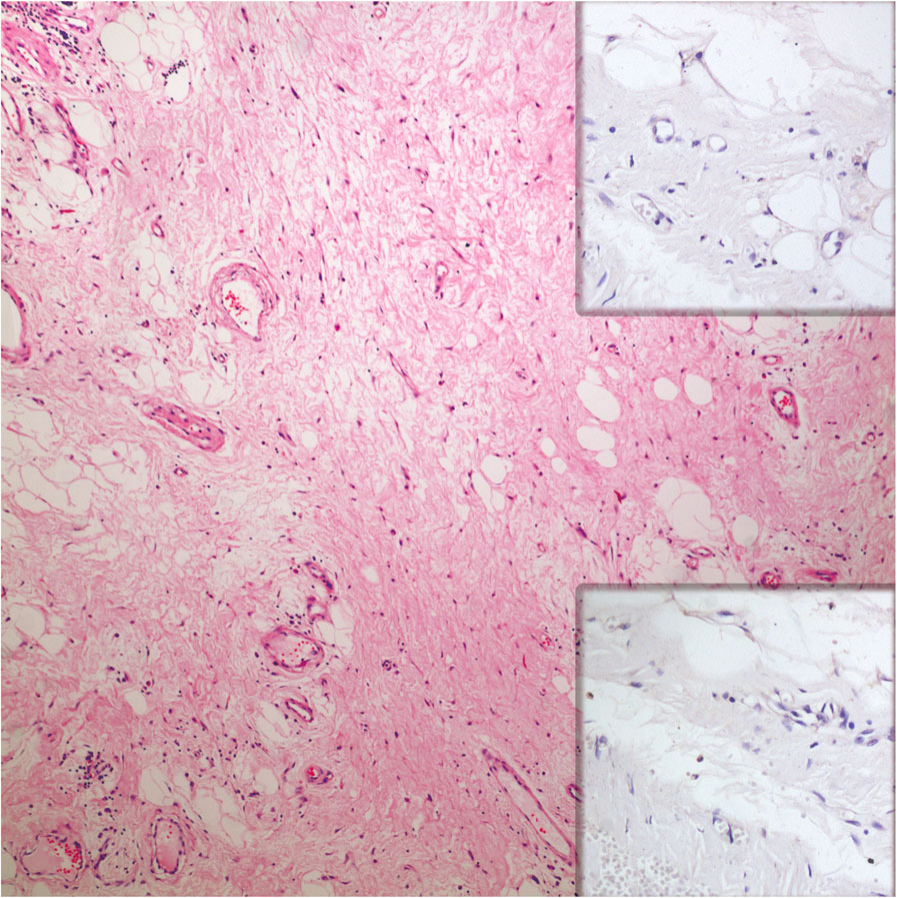
Hypocellular fibrous component containing increased small vessels (H-E × 100). Insets show negative immunostainings for MDM2 and CDK4 (*Upper inset:* Immunohistochemistry, Anti-MDM2 Ab × 200; *Lower inset:* Immunohistochemistry, Anti-CDK4 Ab × 200)

**Fig. 5:**
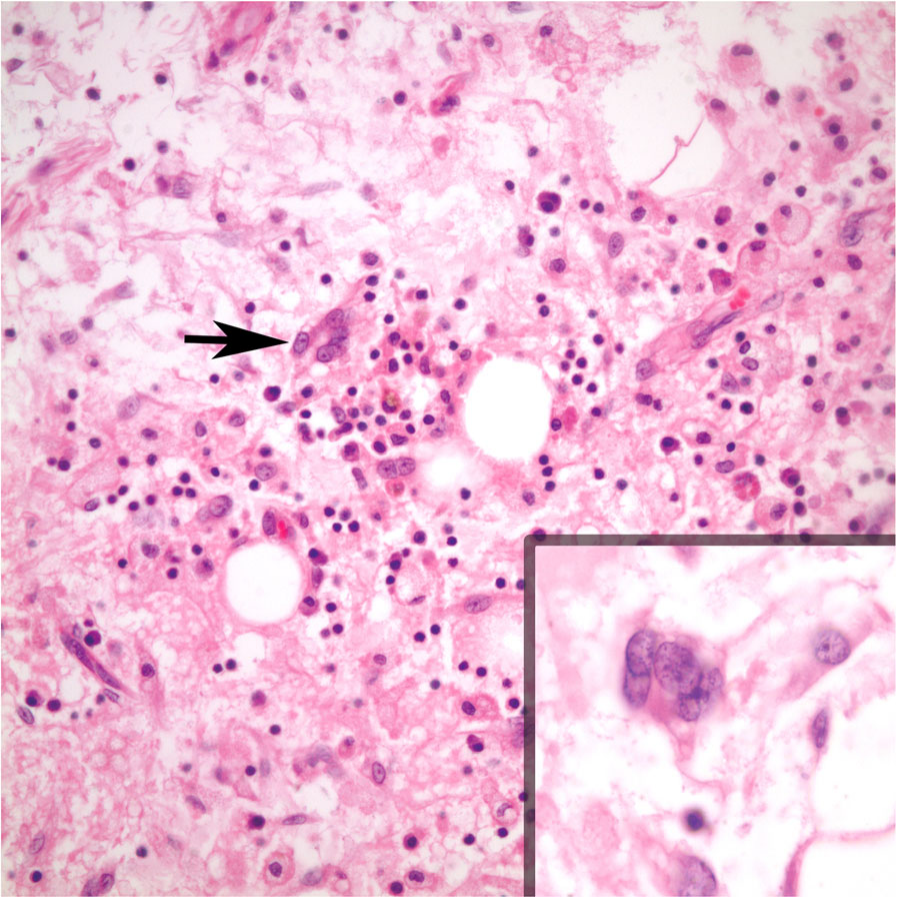
Rare fibroblasts with multilobated nuclei (H-E × 400 with inset: H-E × 1000)

Macroscopically, the tumor was 16x13x6 cm in size, fatty in appearance marbled with irregular whitish solid areas or fibrosis (Fig. 1). Entrapped ureter was stenotic proximally and distally, but dilated at its middle part. Kidney was hydronephrotic with thinned out atrophic parenchyma. A few small stones were detected inside the collecting system. Microscopic examination of the tumor showed mature fat tissue which was devoid of normal architecture owing to expanded interstitial spaces either because of intense edema (Fig. 2) or irregular fibrous streaks (Fig. 3). Fibrous septa between fat lobules contained mildly increased numbers of stromal fibroblasts, fine collagen, vascular proliferation, multifocal lymphocytic infiltration, occasional lymphoid follicles and foamy histiocytes (Fig. 4). Plasma cells were rare, and immunohistochemistry showed only < 2% IgG4+/ IgG+ plasma cell ratio. There were also scattered smooth muscle bundles usually in close association with vessels. Ectatic branching lymphatic channels were not noted. There were neither lipoblasts nor significant cellular atypia. A few scattered fibroblasts carried multilobulated large nuclei which were a bit worrisome (Fig. 5), but these were rare and displayed regular chromatin distribution without hyperchromasia, thus thought to be reactive rather than neoplastic. Additionally, the immunohistochemistry for MDM2 and CDK4 gave negative results (insets-Fig. 4) as well as stains for pan-keratins, HMB-45 and melan-A. Fluorescent in-situ hybridization analysis did not show *MDM2* amplification. The findings were found compatible with MLL. The most possible predisposing factor in this current case appears to be operational trauma which occurred 17 years ago. He did not have lymphedema in the scrotum, legs, abdominal wall, or in the other regions of the body. The patient has stayed recurrence-free for the past 5 years after diagnosis.

## Discussion

The vast majority of adults with MLL are morbidly obese or obese individuals. Kurt H, et al. have reported the largest series of MLL in the literature consisting of 46 patients and the mean body-mass index of their patients was 59.6 kg/m^2^ [2]. As a result, it is suggested that the primary risk factor is obesity and related metabolic syndrome for the development of this disease. The excess adipose tissue is assumed to cause prolonged obstruction of lymphatic vessels or localized ischemia because of traction, leading to massive edema localized mostly in the lower extremity [3–5]. Anterior abdominal wall is the second most common location [2]. The reported other sites include the suprapubic region, mons pubis, vulva, inguinoscrotal, perianal region, penis, and arm [1, 2, 6–10]. The presence of hypothyroidism in some patients suggests an alternative pathogenesis [8]. Trauma and surgery have been counted as common precipitating factors. Several cases of MLL occurring in the normal weight individuals without associating known risk factors such as prior surgery, trauma, and radiotherapy has also been listed in one series [9]. While a large and deep soft tissue mass is often presumed to be malignant in nature, it is worth noting that MLL is one of exceptions, and the current case described above shows that it can even be retroperitoneal.

The histopathology of MLL is rather characteristic although its differential diagnosis may include a variety of other benign and more importantly a few malignant processes. The diagnosis can be straightforward in particular when the mass is located superficially and associated with classic skin alterations. If located in the deep sites of the body as in our case, atypical lipomatous tumor/well-differentiated liposarcoma (ALT/WDLS) will be the most significant differential diagnosis clinically and pathologically. This is especially true if atypical looking spindle cells or multinucleated fibroblasts are present in the fibrous septa similar to those seen in atypical lipomatous tumor/well-differentiated liposarcoma. ALT/WDLS is composed of a relatively mature-looking adipocytic proliferation although significant variation in cell size is easily appreciable. Focal adipocytic nuclear atypia and hyperchromasia contribute to the diagnosis of ALT/WDLS, and scattered hyperchromatic as well as multinucleated stromal cells are often identified within the fibrous septa. Bizarre polylobated hyperchromatic stromal cells of liposarcoma usually contrasts with multilobated reactive stromal fibroblasts in MLL that show open, relatively pale chromatin. Atypical reactive stromal cells in the perinephric soft tissue adjacent to the kidney have also been described in cases of renal cell carcinoma [11]. Kurt H, et al. [2] have noted that dystrophic microcalcifications can mimic hyperchromatic nuclei in some MLL cases. In fact, whenever there is retroperitoneal or deeply seated bland looking lipomatous mass, regardless how convincing the histology is, it is wise to advise that ancillary studies are required for accurate classification and to exclude liposarcoma. FISH for MDM2 or CDK4 gene amplification is considered as the gold standard for diagnosis of WDLS. Immunohistochemistry for MDM2 and CDK4 proteins has been approved as a surrogate method because of its high concordance rate with FISH.

Immunoglobulin G4-related disease (IgG4-RD) and idiopathic retroperitoneal fibrosis can be other considerations in the differential diagnosis of retroperitoneal MLL. IgG4-RD is associated with infiltration of the tissue by numerous IgG4-positive plasma cells as well as storiform fibrosis and obliterative phlebitis. Affected patients in IgG4-RD often have high levels of serum IgG4. Because of their good response to glucocorticoid, correct diagnosis is important in their treatment to avoid unnecessary primary surgery.

Progression of untreated MLL to angiosarcoma has been observed in 10.3% of cases reported in the literature [10]. However, they generally seem to have an excellent prognosis with only occasional recurrences. Nevertheless, they usually reach large sizes. Awareness of this entity is critical to proceed with relevant clinicopathological correlation and separate it from various mimicking conditions. It is essential to remember that MLL can occur in non-obese individuals and in deep places including retroperitoneum. In such circumstances, history of trauma or previous surgery, which may be quite distant must be explored. This will be critical to accommodate the accurate diagnosis and therapeutic approach.

## Conclusions

Massive localized lymphedema has recently emerged as a separate clinical entity. It is characterized by a benign large mass that grows over the years and is usually found in the lower extremity of morbidly obese patients. However, it may develop in unusual sites such as retroperitoneum in non-obese individuals particularly after major surgery. Since gross appearance and histology of MLL are similar to well-differentiated liposarcoma, awareness of its existence is essential for correct diagnosis and patient management.

## Abbrevıatıons

ALT/WDLS: Atypical lipomatous tumor/well-differentiated liposarcoma.
IgG4-RD: Immunoglobulin G4-related disease.
MLL: Massive localized lymphedema.
USG: Ultrasonography.
